# Imaging of brown tumours: a pictorial review

**DOI:** 10.1186/s13244-019-0757-z

**Published:** 2019-07-29

**Authors:** Cheng Xie, Maria Tsakok, Nia Taylor, Karen Partington

**Affiliations:** 1 Department of Radiology, John Radcliffe Hospital, Oxford University Hospital Trust, Headley Way, Headington, Oxford, OX3 9DU UK; 2 Department of Radiology, Churchill Hospital, Oxford University Hospital Trust, Old Road, Headington, Oxford, OX3 7LE UK; 3 Department of Radiology, Nuffield Orthopaedic Centre, Oxford University Hospital Foundation Trust, Windmill Road, Headington, Oxford, OX3 7HE UK

**Keywords:** Brown tumour, Parathyroid adenoma, Hyperparathyroidism, Parathyroid ultrasound, Sestamibi

## Abstract

Brown tumours do not represent neoplastic process, but they are focal bony lesions due to bone remodelling from either primary or secondary hyperparathyroidism. Their incidence is also low. The current literature on brown tumour is mainly in the form of case reports that focus on single affected sites. This pictorial review describes the full imaging workup and pathway of suspected brown tumour in the setting of both primary and secondary hyperparathyroidism. It aims to illustrate the management strategy to aid both clinicians and radiologists in suspected cases of brown tumour. We highlight the complementary roles that different imaging modalities can play in different settings including the importance of parathyroid ultrasound, ^99m^Tc-sestamibi scintigraphy and SPECT/CT in the localisation of the parathyroid adenoma. We present cases with full clinical and imaging workup in both the acute and chronic setting and scenarios that require exclusion of primary and secondary bone malignancies.

## Key points

- Primary hyperparathyroidism from parathyroid adenoma is the most common cause of brown tumours.
- Imaging workup for suspected cases should include ultrasound, ^99m^TC-sestamibi scintigraphy or SPECT/CT.
- 4D-CT can also aid the localisation of parathyroid adenoma.
- Sclerotic bone lesions are most common but lytic lesion is also possible.
- It is important to consider differential diagnosis including primary and secondary bone tumours.

## Introduction

The name brown tumour originated from their gross histological appearance being a brownish mass consisted of a combination of recurrent micro-fractures at various stages of remodelling with blood, haemosiderin, fibrous and connective tissue [1]. Unlike the name suggests, brown tumours are not the result of a neoplastic process. They are focal bony lesions as a consequence of bone remodelling from either hyperparathyroidism or paraneoplastic syndrome [2–5].

The condition is more frequently seen in women than in men, and present in the fifth to sixth decade of life. The most common cause of primary hyperparathyroidism is parathyroid adenoma causing the hypersecretion of parathyroid hormone (PTH) [3]. Secondary hyperparathyroidism is usually seen in end-stage renal failure [2, 4–6]. From a biochemical perspective, the main difference is that primary hyperparathyroidism causes an increase in serum calcium and reduced phosphate. Secondary hyperparathyroidism produces the opposite—hypocalcaemia and hyperphosphataemia. Symptomatically, patients suffering from primary hyperthyroidism are more likely to present with bone pain, renal stones, gastrointestinal and neurological complaints commonly recognised as “bones, stones, groans, and moans” [7]. Malignant tumours can also produce these symptoms as part of the paraneoplastic syndrome, and it is due to high levels of parathyroid hormone-related peptide (PTHrP) mimicking the effect of PTH [8]. In these cases, brown tumours could be mistaken for bone metastases [9–11].

Disease presentation with brown tumour is rare. The reported incidence of brown tumour has been 3% and 1.5% in primary and secondary hyperparathyroidism, respectively [7, 12]. Its incidence as the result of the paraneoplastic syndrome has not been previously reported. Published literature on brown tumour is mainly in the form of case reports describing a single affected site [1, 5, 8, 9, 11–16]. The objective of this pictorial review is to illustrate the various presentations of brown tumour (both primary and secondary hyperparathyroidism), its full imaging workup and characteristics of the tumour through a case series.

## Imaging of the parathyroid gland

For parathyroid imaging in patients with suspected hyperparathyroidism, ultrasound and ^99m^Tc-sestamibi scintigraphy are the most commonly used imaging modalities. Four-dimensional computed tomography (4D-CT) is an alternative imaging modality used in the evaluation of hyperparathyroidism.

**Fig. 1:**
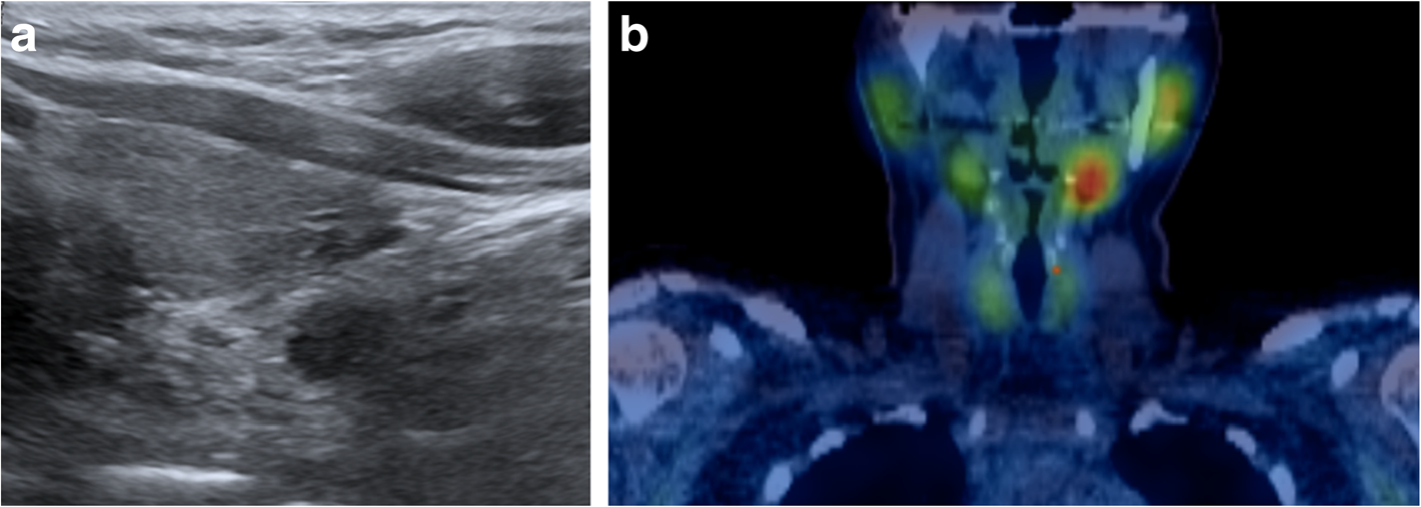
**a**, **b** Ultrasound and coronal view SPECT/CT of the thyroid and hyperparathyroid in the 32-year-old female patient showed a 9-mm hypoechoic nodule with high uptake in the area of the left inferior parathyroid and localising a parathyroid adenoma

^99m^Tc-sestamibi scintigraphy can help confirm the presence and location of a parathyroid adenoma. Both the thyroid and parathyroid glands will demonstrate tracer uptake on the initial images. However, on the delayed images usually taken at 2 h post-administration of the radioactive tracer, a parathyroid adenoma shows persistent avid uptake due to its delayed washout of the tracer compared to the thyroid gland (Fig. 1).

Ultrasound is commonly used for patients with suspected parathyroid adenoma and used to assess the feasibility of minimally invasive surgery. The adenoma is frequently identified as a homogeneously hypoechoic lesion overlying the thyroid gland (Fig. 1). The lower pole of the thyroid should be closely examined, as the parathyroid adenoma is commonly found inferior, posterior or lateral to this position. The entire thyroid gland should be reviewed as the superior parathyroid gland can be found in the upper or mid pole of the thyroid [17].

Hybrid imaging using single-photon emission computed tomography (SPECT) fused with the corresponding computed tomography (CT) is the preferred three-dimensional functional imaging for the localisation of parathyroid tumour [18]. The addition of arterial phase contrast enhancement during CT also helps with the localisation of the adenoma. This would be particularly useful for cases in which the adenoma is sestamibi negative or during ultrasound when the artefact in trachea/oesophagus can obscure a posterior lesion. Both ultrasound and SPECT/CT examinations improve the localisation accuracy of the parathyroid tumours [19].

4D-CT increasingly been used for pre-operative localisation of parathyroid adenoma [20]. The multiplanar nature of CT offers three-dimensional anatomical assessment, and combined with the functional aspect the adenoma’s contrast enhancement pattern provides the fourth dimension. A typical parathyroid adenoma has low attenuation on the pre-contrast images and, subsequently, shows arterial enhancement and washout during venous/delayed phase. The adjacent thyroid tissue has higher attenuation on the pre-contrast images due to the high attenuation iodine. The thyroid tissue also shows persistent contrast enhancement during venous/delayed phase. The parathyroid adenoma arterial enhancement help differentiate it from a lymph node, which shows peak enhancement at the delayed phase [21–25]. In addition, the supplying artery or draining vein of the parathyroid adenoma is usually tortuous and distended, which can sometimes be identified on the 4D-CT as the “polar vessels” sign [26]. Recent studies that directly compared 4D-CT with sestamibi SPECT/CT in pre-operative localisation of parathyroid adenoma showed that 4D-CT provided between pre-operative localisation in patients with solitary adenomas and multigland disease [27, 28].

## Imaging of skeletal involvement

Clinically, brown tumours present as a slow growing palpable bony swelling and may cause bone pain or pathological fractures. It is also possible to present with symptoms of weakness, weight loss, polyuria and recurrent stone formation associated with the hyperparathyroidism [12]. The bone pain from the brown tumour is also comparatively milder than the radiological findings and malignant bone lesions [11]. As a result, brown tumours are often incidental findings on plain radiograph or computed tomography (CT) for another indication. Dedicated CT or magnetic resonance imaging (MRI) is often performed to assess the full extent of musculoskeletal involvement and to guide a bone biopsy if necessary. Previous case reports have identified that brown tumours are preferentially located in the mandible, ribs, clavicle and pelvis and rarely seen in the skull or associated with the nasal/paranasal sinuses [1, 5, 8, 9, 11–16]. The radiological features of browns tumours in the reported cases mainly consisted of single or multiple well-defined osteolytic lesions, single or multi-lobular, usually with bone expansion and can demonstrate bony destruction and be associated with pathological fractures. There is also variability in its radiological characteristics, which can include an ill-defined lesion, mixed lytic/sclerotic lesion or margin, with adjacent soft tissue involvement [11, 29, 30]. In these situations, a malignant lesion should be considered in the differential diagnosis and histologic confirmation is recommended. Further correlation with hypercalcaemia and hyperparathyroidism is also important because histologic differentiation between brown tumour and other giant cell-containing lesions can be difficult [31–33].

## Patient presentation and imaging workup

**Fig. 2:**
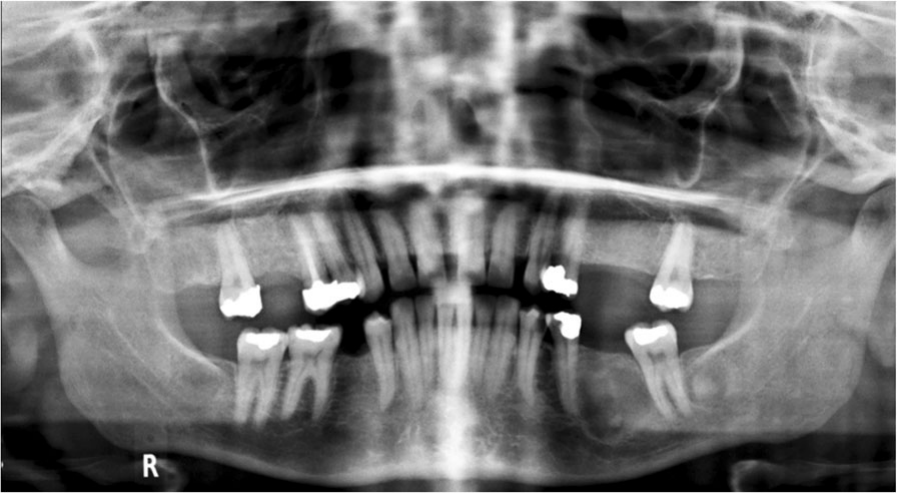
On the orthopantomogram (OPG), there is a single well-defined, corticated, lucent lesion in the body of the left mandible between the premolar and only remaining molar tooth

**Fig. 3:**
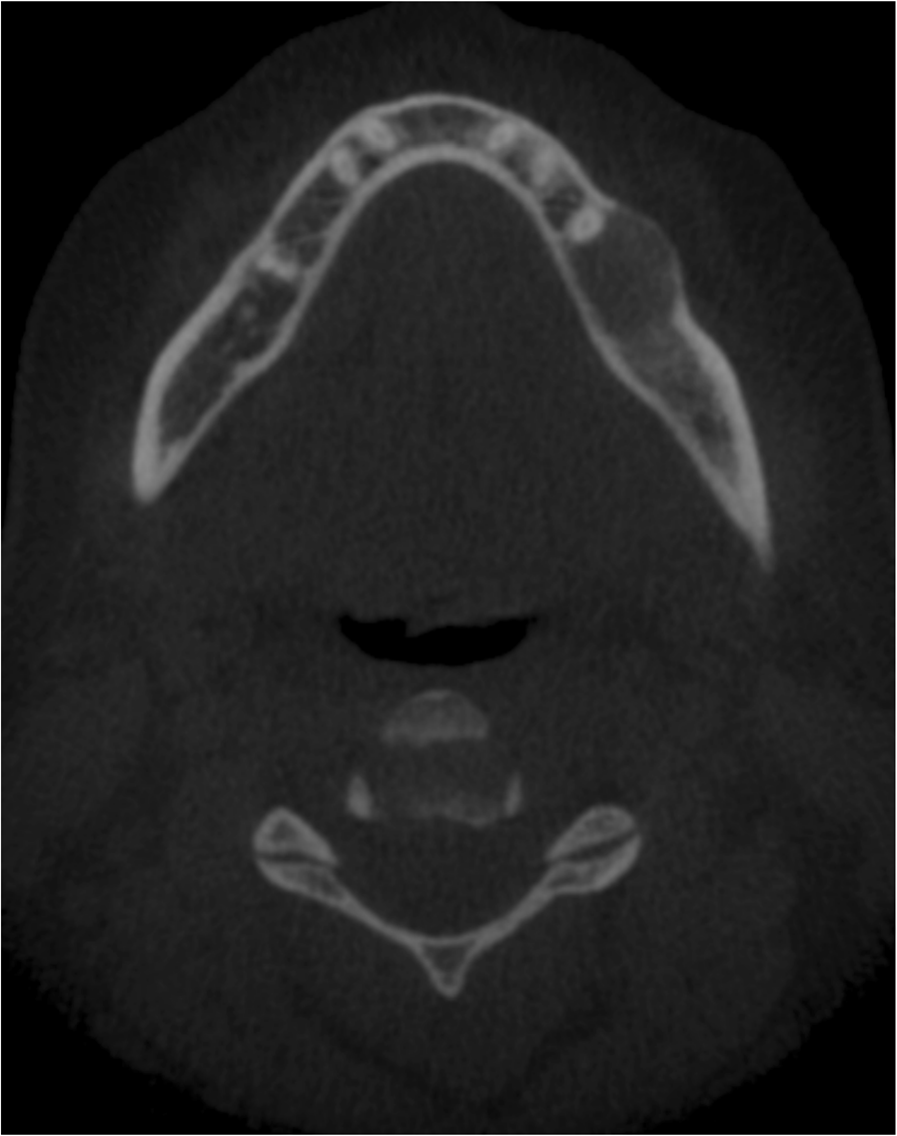
CT of the same patient showed a solitary expansile left mandibular low density lesion with associated cortical thinning

Our first patient is a previously healthy 32-year-old female who presented with a 6 months history of intermittent pain and swelling at the left jaw. An ultrasound of the salivary glands excluded any obstructive calculus. The orthopantomogram (OPG) showed poor dentition with several absent premolar and molar teeth. Most importantly, there was a single well-defined, corticated, lucent lesion in the body of the left mandible between the premolar and only remaining molar tooth (Fig. 2). The subsequent CT confirmed a solitary expansile left mandibular low density lesion with associated cortical thinning (Fig. 3). The patient’s blood results revealed elevated parathyroid hormone (PTH) 12.8 pmol/L (normal range 1.3–7.6 pmol/L) and normal corrected calcium and phosphate levels. The differential diagnoses include both odontogenic causes (primordial odontogenic keratocyst, residual cyst, ameloblastoma), and non-odontogenic causes, which in this setting of elevated PTH, a brown tumour was the most likely cause. In order to confirm the presence of parathyroid adenoma, parathyroid ultrasound and ^99m^Tc-sestamibi SPECT/CT were performed. On ultrasound, there was a 9-mm hypoechoic nodule posterior to the inferior left lobe of the thyroid (Fig. 1), and SPECT/CT showed residual high uptake in the corresponding area to localise the left inferior parathyroid adenoma (Fig. 1). Both biochemical and imaging findings supported the diagnosis of a brown tumour in the left mandible.

**Fig. 4:**
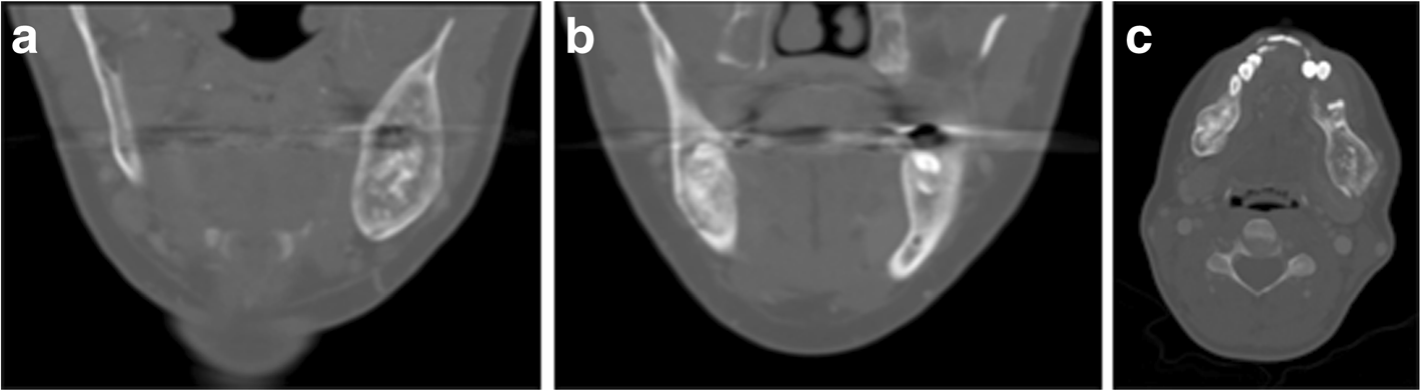
**a**–**c** 31-year-old female patient with bilateral jaw pain. CT showed bilateral mandibular brown tumours. Lesions contained stipple calcification

Brown tumour in the mandible can also be bilateral and cause symptoms on both sides. Our second patient was a 31-year-old lady who presented with bilateral jaw pain and undergone the same imaging pathway, which showed bilateral mandibular brown tumours from underlying hyperparathyroidism (Fig. 4). The unique imaging feature in this case was that the mandibular lesions contained stipple calcification.

**Fig. 5:**
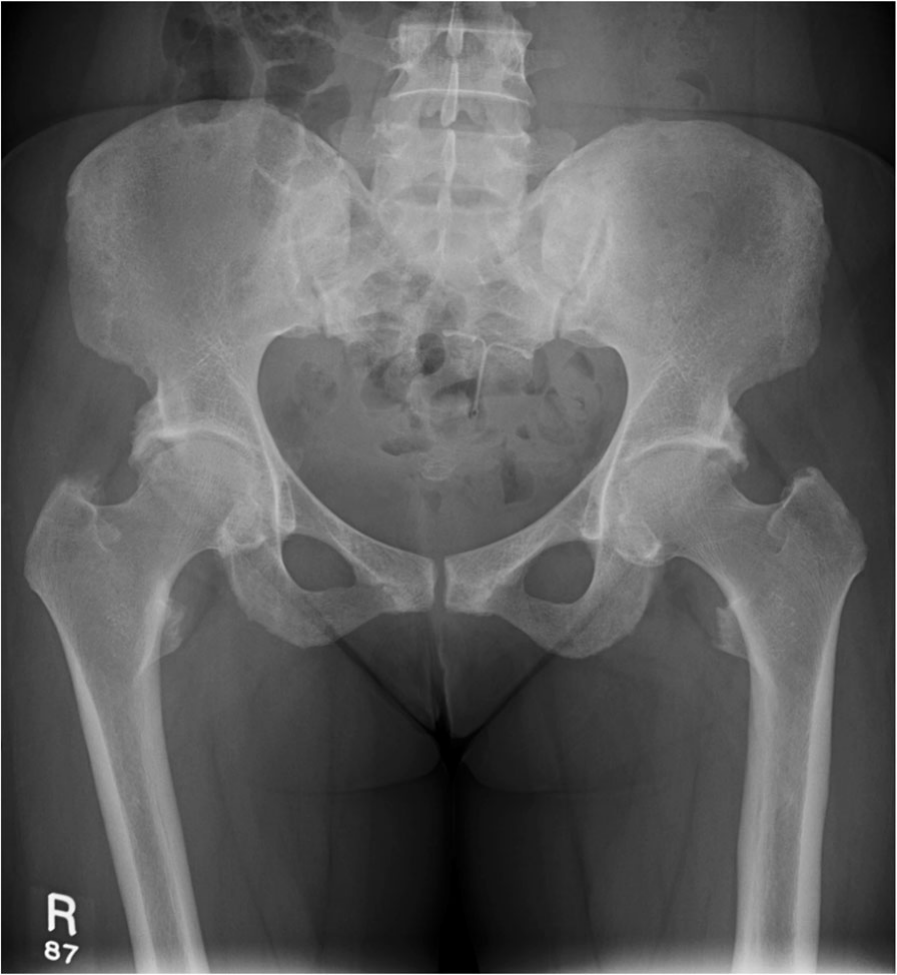
The pelvic radiograph showed mild degree of osteoarthritis in both hip joints with femoro-acetabular impingement (cam and pincer impingement)

**Fig. 6:**
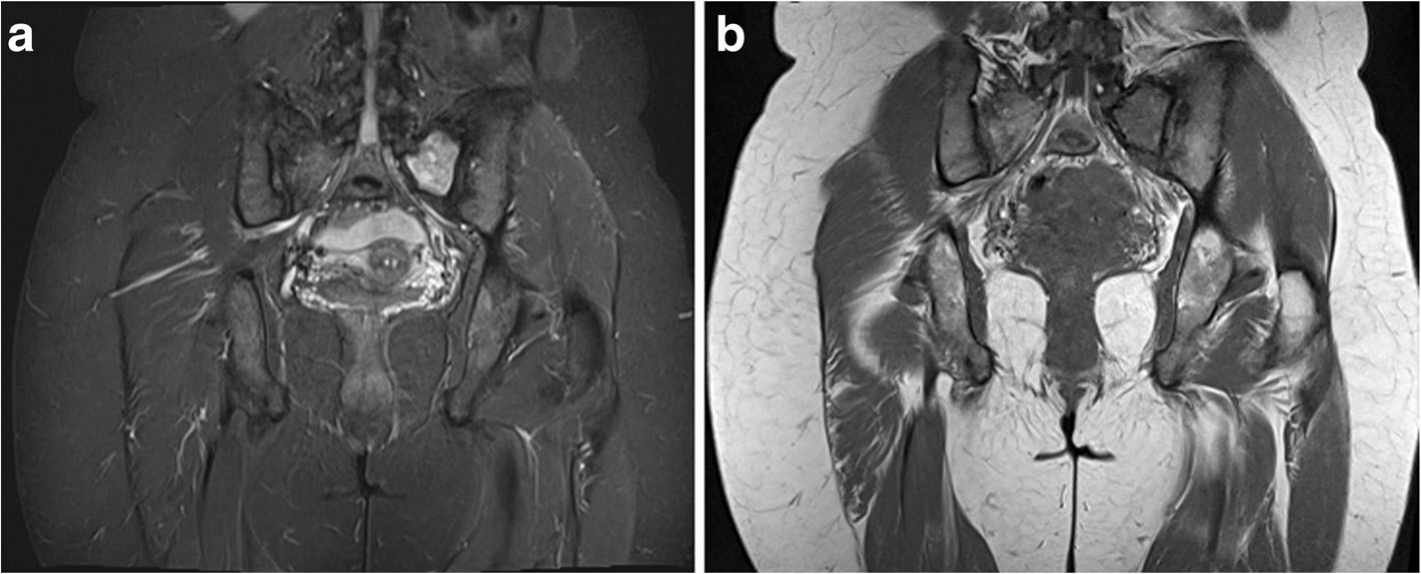
**a**, **b** MRI of the same patient showed an incidental lesion in the left sacral ala—well-defined with high T2 and intermediate T1 signal

**Fig. 7:**
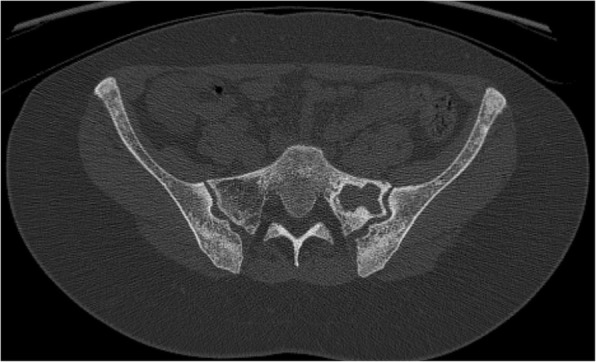
CT of the patient showed a well-corticated lucent lesion with patchy high density areas in the rest of the pelvic bone in keeping with previous rickets

**Fig. 8:**
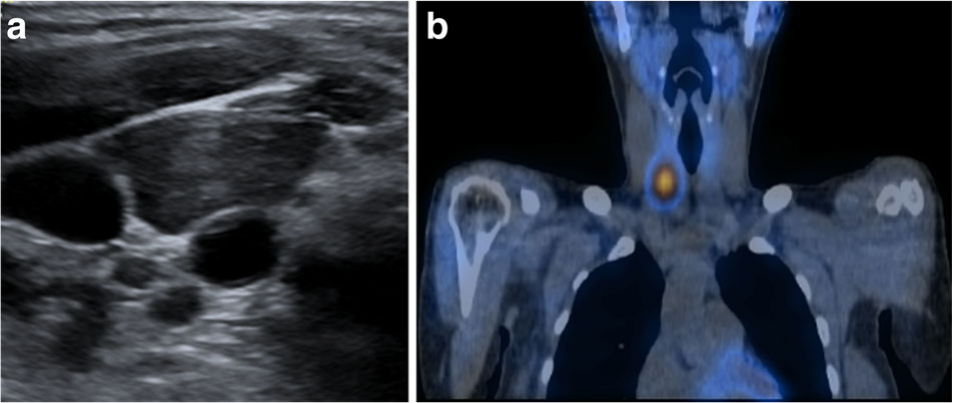
**a**, **b** Ultrasound and SPECT/CT showed a 15-mm right inferior parathyroid adenoma with residual high uptake

Our next patient was a 40-year-old female who had rickets as a child. She presented with worsening of chronic bilateral hip pain over the last 3 months. This patient’s pelvic radiograph (Fig. 5) showed a mild degree of osteoarthritis in both hip joints with femoro-acetabular impingement (a mixture of cam and pincer impingement). The tendon insertion sites around the pelvis in particular the greater and lesser trochanters and inferior pubic rami were less well-defined with new bone formation consistent with enthosopathy. Further MRI to investigate the hip pain found an incidental lesion in the left sacral ala. It was well-defined with high T2 and intermediate T1 signal (Fig. 6). CT also showed that it was a well-corticated lucent lesion with patchy high density areas in the rest of the pelvic bone in keeping with previous rickets (Fig. 7). Bloods results revealed mildly elevated corrected calcium 2.64 mmol/L (normal range 2.1–2.6 mmol/L), reduced phosphate 0.34 mmol/L (normal range 0.7–1.45 mmol/L) and markedly raised PTH level of 31.6 pmol/L. The suspicion of primary hyperparathyroidism was confirmed on ultrasound and SPECT/CT, which showed a 15-mm right inferior parathyroid adenoma with persistent high uptake (Fig. 8). The lesion in the left sacral ala was diagnosed as a brown tumour in light of the clinical and imaging findings.

**Fig. 9:**
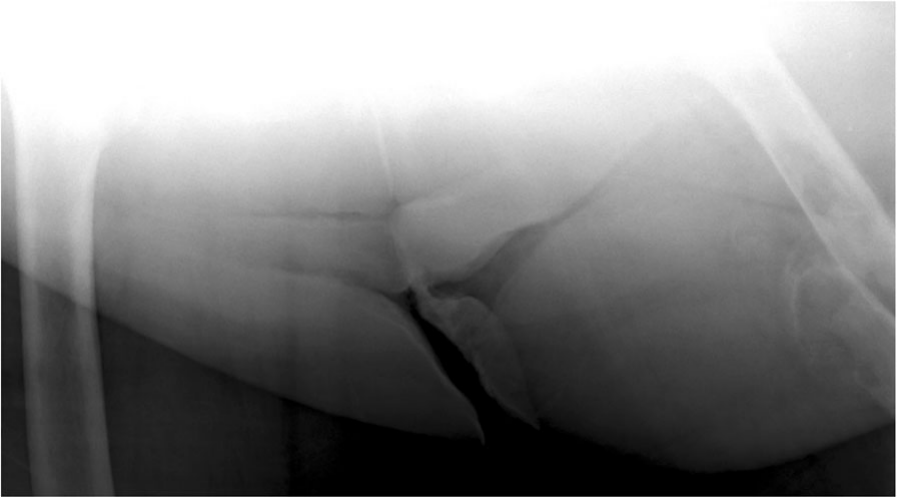
Patient presented with left upper leg pain without history of trauma. The pelvic radiograph showed a displaced fracture of the left femoral shaft with associated lytic lesions

**Fig. 10:**
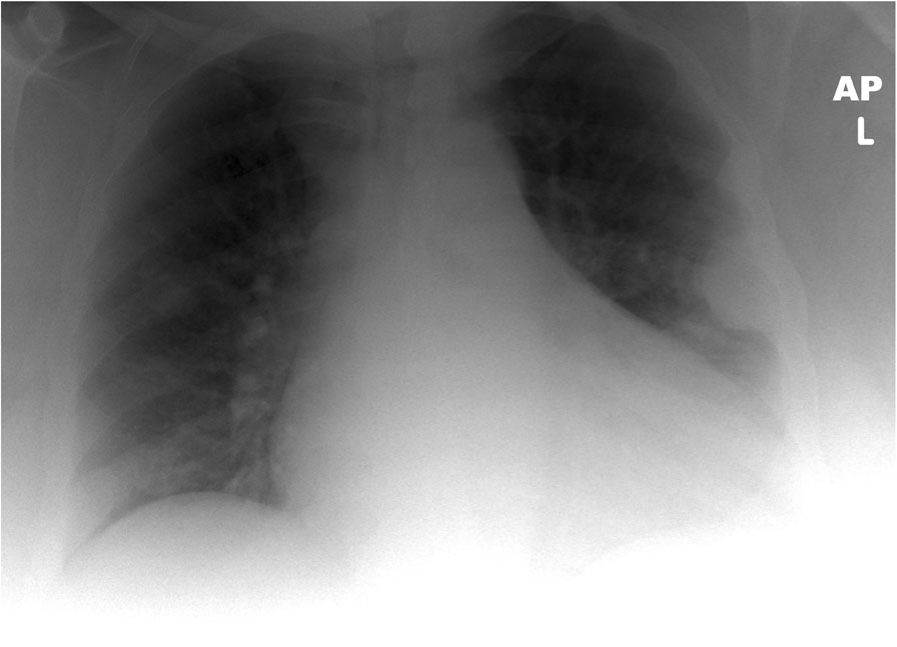
Chest radiograph of the same patient showed sclerotic expansion of the left lateral ribs

**Fig. 11:**
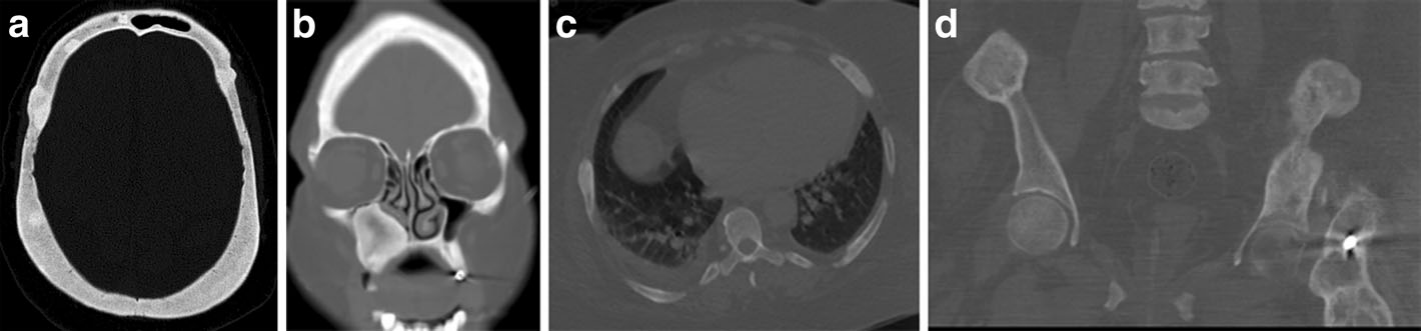
CT showed multiple expansile mixed lytic/sclerotic lesions in the calvarium, right maxillary antrum, ribs and iliac crests bilaterally

**Fig. 12:**
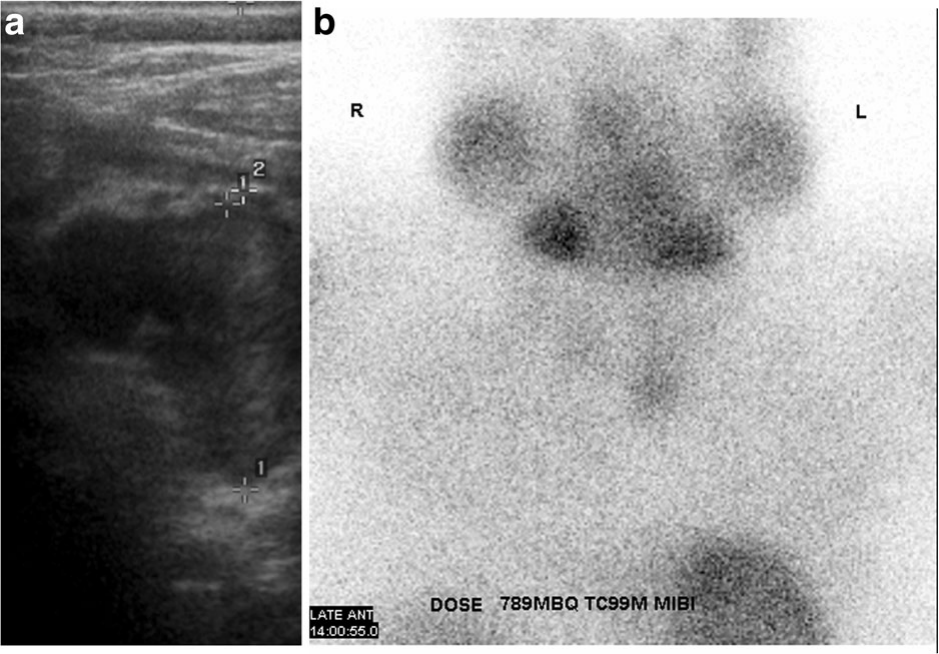
**a**, **b** Ultrasound and ^99m^Tc-sestamibi SPECT showed a 2.5-cm left inferior parathyroid adenoma

Apart from chronic pain and/or hard swelling, patient with brown tumour could also present with acute pathological fractures. A 57-year-old male patient presented with sudden onset of left upper leg pain without history of trauma. The patient’s pelvic radiograph showed a displaced fracture of the left femoral shaft with associated lytic lesions at the fracture site (Fig. 9). In addition, the chest radiograph showed sclerotic expansion of the left lateral ribs (Fig. 10). The initial working diagnosis was a pathologic fracture due to metastatic disease with further bone metastasis in the ribs. As a result, a CT of the head and body was performed to investigate a primary malignancy. In addition, the prostate was examined for the possibility of prostate cancer. The CT showed multiple expansile mixed lytic/sclerotic lesions in the calvarium, right maxillary antrum, ribs and iliac crests bilaterally (Fig. 11). No thoracic, intra-abdominal or prostate abnormality was found. Patient’s blood results demonstrated normal prostate-specific antigen (PSA), but markedly elevated PTH 182 pmol/L, mildly raised corrected calcium of 2.9 mmol/L and reduced phosphate of 0.68 mmol/L. These findings make malignancy the less likely cause and suggest primary hyperparathyroidism the more likely cause of the bone lesions. Primary hyperparathyroidism was confirmed when the ultrasound and ^99m^Tc-sestamibi SPECT (before the advent of SPECT/CT) showed a 2.5-cm left inferior parathyroid adenoma (Fig. 12).

**Fig. 13:**
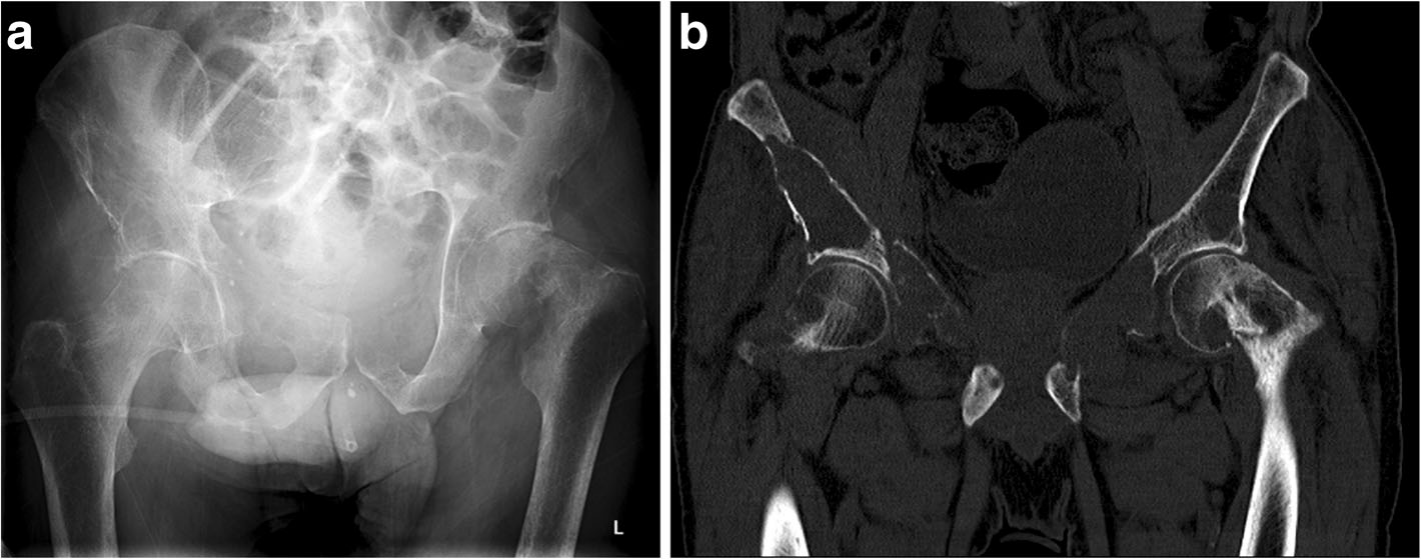
**a**, **b** Patient presented after a fall. The pelvic radiograph showed a left subcapital neck of femur fracture with a lytic area in the inferior aspect of the femoral head. Coronal CT showed multiple lytic areas

**Fig. 14:**
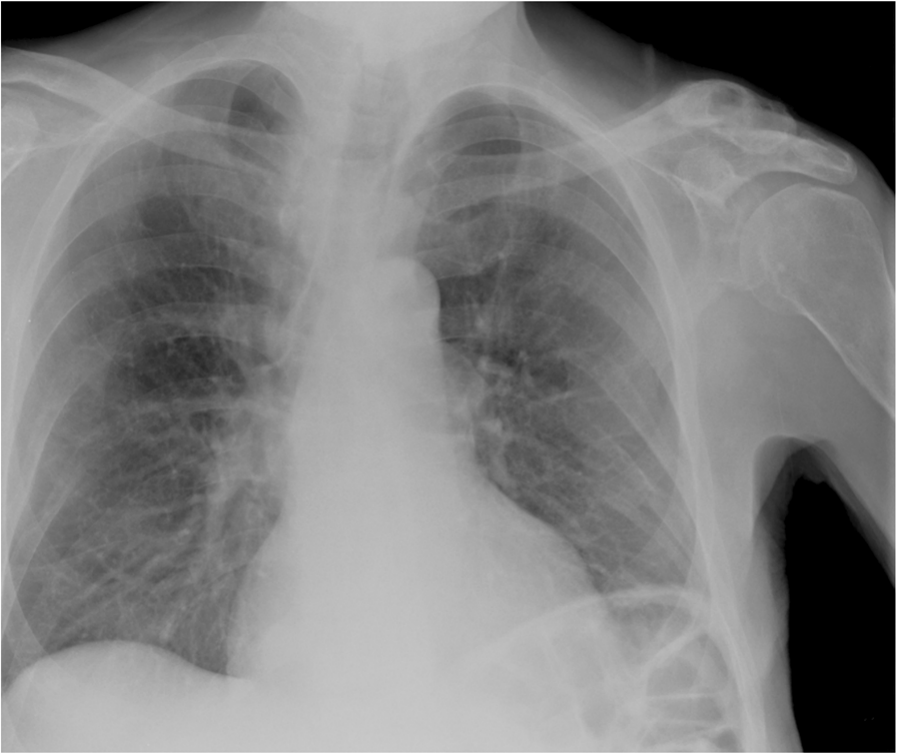
Chest radiograph of the same patient showed an expansile mixed lytic/sclerotic lesion in the lateral aspect of the left clavicle

In the above case, the multiple brown tumours were mixed lytic/sclerotic, and some of lesions showed sclerotic rim. It is important to highlight that the lesions can also be lytic or a mixture of both. The following 69-year-old male patient with known primary hyperparathyroidism and awaiting parathyroidectomy presented after a fall. The pelvic radiograph showed a left subcapital neck of femur fracture with a lytic area in the inferior aspect of the femoral head (Fig. 13). Close inspection of the right hip joint showed additional lytic areas in the right acetabulum and the ilium, which were also delineated on the subsequent CT (Fig. 13). Patient chest radiograph showed an expansile lytic/sclerotic lesion in the lateral aspect of the left clavicle (Fig. 14). The multiple brown tumours in this case were not only mainly lytic lesions, but also mixed in the left clavicle.

**Fig. 15:**
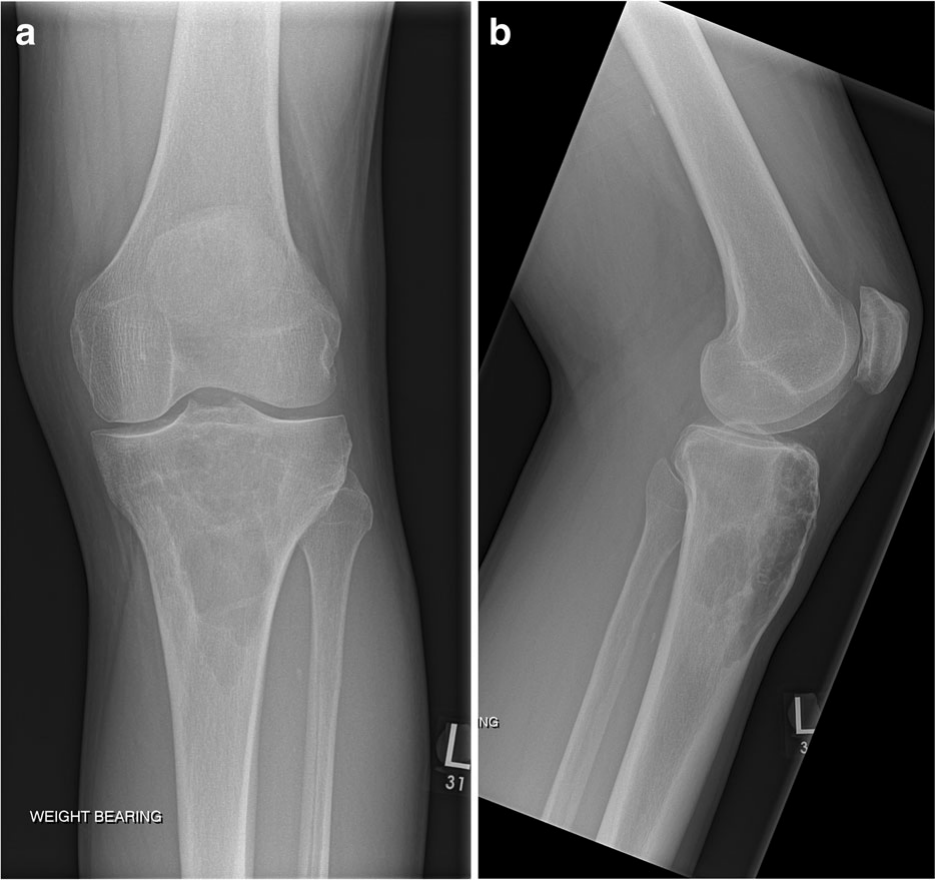
**a**, **b** The plain film of his left knee showed a multiloculated lytic lesion in the proximal tibial metaphysis close to the cortical surface

**Fig. 16:**
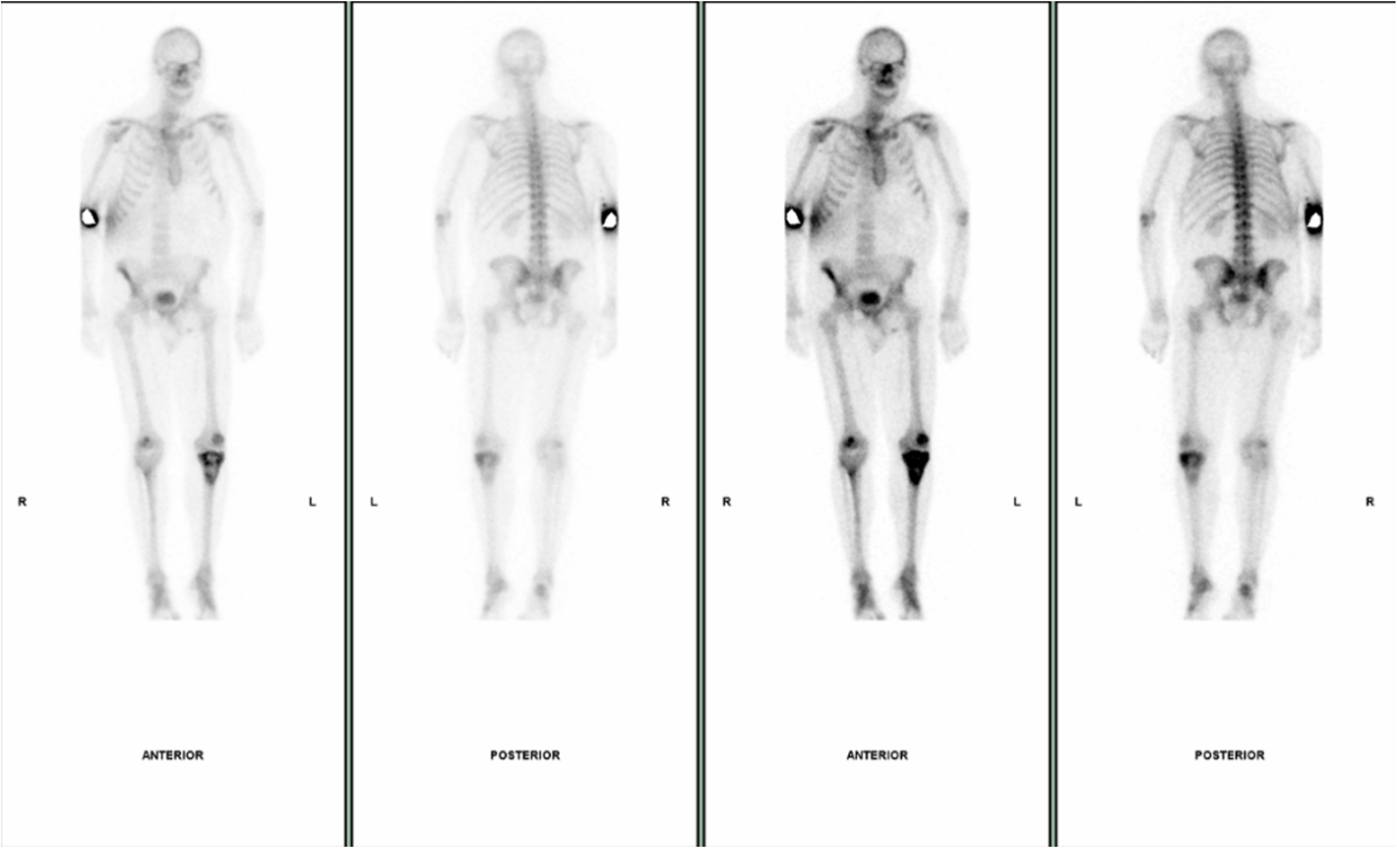
Whole body bone scan demonstrated a single lesion with elevated uptake in the left tibia metaphysis

**Fig. 17:**
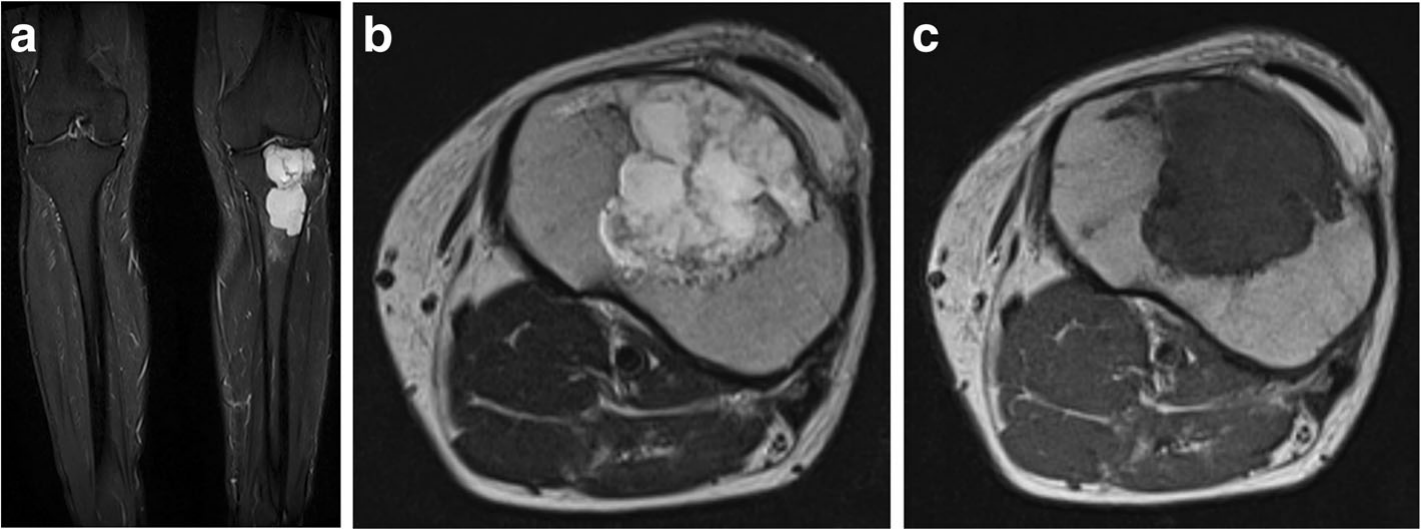
**a**–**c** MRI showed a multiloculated lesion with high T2, and Short-Tau Inversion Recovery (STIR) signals. The lesion is predominately a low T1 with mildly hyperintense patchy areas, which suggested intralesional haemorrhage. No fluid/fluid levels

The next case highlights the importance of not to make immediate assumptions that bone lesions in patients with hyperparathyroidism are always brown tumours. This 54-year-old male patient, who was a keen runner, presented with chronic left knee pain. The plain film of his left knee showed a multiloculated lytic lesion in the proximal tibial metaphysis close to the cortical surface. There was no associated cortical breach or periosteal reaction (Fig. 15). His blood results showed elevated PTH of 8.3 pmol/L and mildly elevated corrected calcium of 2.61 mmol/L and normal phosphate level. The initial working diagnoses included giant cell tumour, metastatic deposit, chondrosarcoma and Brown tumour associated with hyperparathyroidism. To help exclude other bone lesions, a whole body bone scan was performed and it demonstrated the single lesion with elevated uptake in the left tibia metaphysis (Fig. 16). The following MRI showed a multiloculated lesion with high T2 and Short-Tau Inversion Recovery (STIR) signals. It was predominately low T1 but consisted of mildly hyperintense patchy areas, which suggested intralesional haemorrhage (Fig. 17). No fluid/fluid levels were present. Given the imaging characteristics, a targeted biopsy of the lesion was performed and histologically confirmed a grade 1 chondrosarcoma. This case illustrates the importance of considering other differential diagnoses including malignant bone tumours in the context of hyperparathyroidism.

**Fig. 18:**
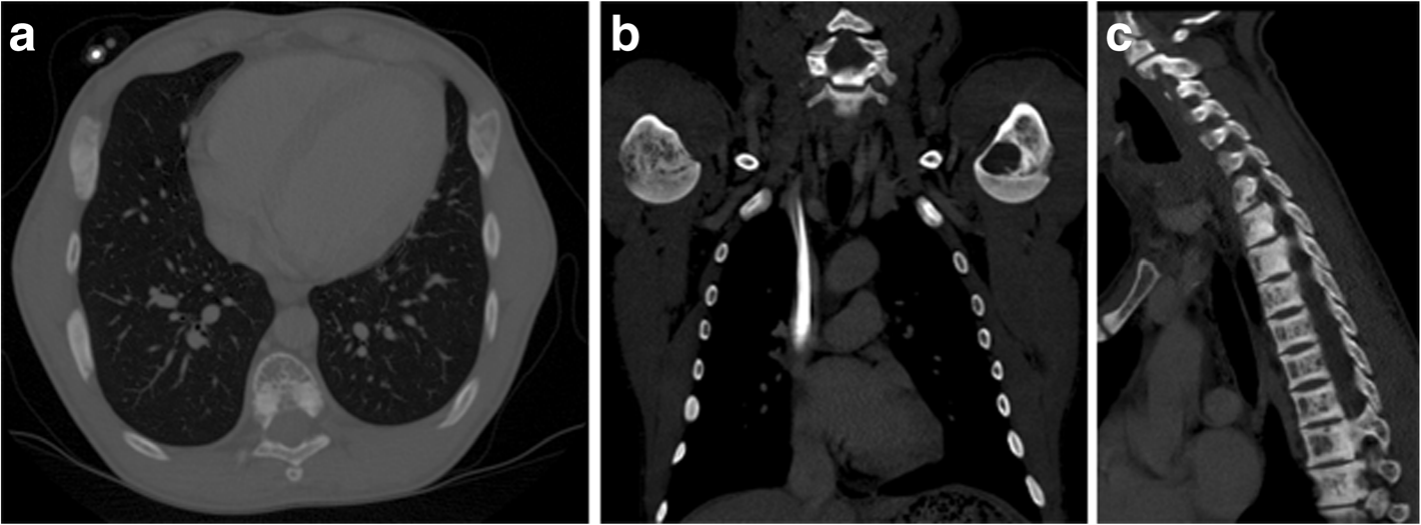
**a**–**c** Patients with secondary hyperparathyroidism due to chronic renal failure. CT demonstrated asymptomatic bilateral mixed lytic/sclerotic expansile rib lesions, lytic lesion in the left humeral head and the rugger-jersey spine

The final two cases were patients with chronic renal failure and receiving long-term haemodialysis. Patients in this category have secondary hyperparathyroidism as a result of their chronic renal disease. CT imaging of these patients demonstrated asymptomatic bilateral mixed lytic/sclerotic expansile rib lesions and lytic lesion in the left humeral head (Fig. 18). These lesions are brown tumours and incidence is lower compared to the association with primary hyperparathyroidism [7–12]. In addition, the vertebral spines undergo bone resorption resulting in centrally lucent vertebral bodies and sclerotic endplates producing the characteristic rugger-jersey spine (Fig. 18). The bone lesions could also affect the orofacial area and result in facial or oral cavity swelling. Both CT and/or MRI would be helpful for diagnosis and management [34, 35].

## Conclusion

Brown tumour is a rare bony disorder as a consequence of hyperparathyroidism. Apart from biochemical investigations for hyperparathyroidism, both US and sestamibi scintigraphy should be used to confirm the location of the active parathyroid gland. Patients can present with systemic symptoms of hyperparathyroidism or insidious pain, bony swelling and pathologic fracture from the brown tumour. The radiologic features of brown tumour can be variable, which can be single or multiple lesions, lytic or a mix of both. In addition to the plain film, CT and/or MRI should be performed to help differentiate from other bone tumours, which should be considered even in the context of hyperparathyroidism. If there is any doubt in the diagnosis, biopsy of the bone tumour is recommended for definitive diagnosis.

## Data Availability

Not applicable

## Abbreviations

4D-CT: Four-dimentional computed tomography
CT: Computed tomography
MRI: Magnetic resonance imaging
OPG: Orthopantomogram
PSA: Prostate-specific antigen
PTH: Parathyroid hormone
PTHrP: Parathyroid hormone-related peptide
SPECT: Single-photon emission computed tomography
STIR: Short-Tau Inversion Recovery

## References

[1] Schweitzer VG, Thompson NW, McClatchey KD (1986). Sphenoid sinus brown tumor, hypercalcemia, and blindness: an unusual presentation of primary hyperparathyroidism. Head Neck Surg.

[2] Marini M, Vidiri A, Guerrisi R, Campodonico F, Ponzio R (1992). Progress of brown tumors in patients with chronic renal insufficiency undergoing dialysis. Eur J Radiol.

[3] Parisien M, Silverberg SJ, Shane E, Dempster DW, Bilezikian JP (1990). Bone disease in primary hyperparathyroidism. Endocrinol Metab Clin North Am.

[4] Rao P, Solomon M, Avramides A et al (1978) Brown tumors associated with secondary hyperparathyroidism of chronic renal failure. J Oral Surg 36(2):154–159 271707

[5] Weiss RR, Schoeneman MJ, Primack W, Rozycki D, Bennett B, Greifer I (1980). Maxillary brown tumor of secondary hyperparathyroidism in a hemodialysis patient. JAMA..

[6] Nassar George M., Ayus Juan Carlos (1999). Brown Tumor in End-Stage Renal Disease. New England Journal of Medicine.

[7] Grulois V, Buysschaert I, Schoenaers J, Debruyne F, Delaere P, Vander Poorten V (2005). Brown tumour: presenting symptom of primary hyperparathyroidism. B-ENT..

[8] Triantafillidou K, Zouloumis L, Karakinaris G, Kalimeras E, Iordanidis F (2006). Brown tumors of the jaws associated with primary or secondary hyperparathyroidism. A clinical study and review of the literature. Am J Otolaryngol.

[9] Hsieh MC, Ko JY, Eng HL (2004). Pathologic fracture of the distal femur in osteitis fibrosa cystica simulating metastatic disease. Arch Orthop Trauma Surg.

[10] Joyce JM, Idea RJ, Grossman SJ, Liss RG, Lyons JB (1994). Multiple brown tumors in unsuspected primary hyperparathyroidism mimicking metastatic disease on radiograph and bone scan. Clin Nucl Med.

[11] Hoshi Manabu, Takami Masatsugu, Kajikawa Michiko, Teramura Kazuhiro, Okamoto Takashi, Yanagida Ikuhisa, Matsumura Akira (2007). A case of multiple skeletal lesions of brown tumors, mimicking carcinoma metastases. Archives of Orthopaedic and Trauma Surgery.

[12] Younes NA, Mahafza WS, Agabi SS (2004). Brown tumor of the femur associated with double parathyroid adenomas. Saudi Med J.

[13] Erem C., Hacihasanoglu A., Cinel A., Önder Ersöz H., Reis A., Sari A., Köse M., Ukinç K., Telatar M. (2004). Sphenoid sinus brown tumor, a mass lesion of occipital bone and hypercalcemia: An unusual presentation of primary hyperparathyroidism. Journal of Endocrinological Investigation.

[14] Guney E, Yigitbasi OG, Bayram F, Ozer V, Canoz O (2001). Brown tumor of the maxilla associated with primary hyperparathyroidism. Auris Nasus Larynx.

[15] Gelman R, Gellad FE (1991). Brown tumor of the facial bones. AJNR Am J Neuroradiol.

[16] Keyser JS, Postma GN (1996). Brown tumor of the mandible. Am J Otolaryngol.

[17] Johnson NA, Tublin ME, Ogilvie JB (2007). Parathyroid imaging: technique and role in the preoperative evaluation of primary hyperparathyroidism. AJR Am J Roentgenol.

[18] Taubman ML, Goldfarb M, Lew JI (2011). Role of SPECT and SPECT/CT in the surgical treatment of primary hyperparathyroidism. Int J Mol Imaging.

[19] Ruda JM, Hollenbeak CS, Stack BC (2005). A systematic review of the diagnosis and treatment of primary hyperparathyroidism from 1995 to 2003. Otolaryngol Head Neck Surg.

[20] Rodgers Steven E., Hunter George J., Hamberg Leena M., Schellingerhout Dawid, Doherty David B., Ayers Gregory D., Shapiro Suzanne E., Edeiken Beth S., Truong Mylene T., Evans Douglas B., Lee Jeffrey E., Perrier Nancy D. (2006). Improved preoperative planning for directed parathyroidectomy with 4-dimensional computed tomography. Surgery.

[21] Bann DV, Zacharia T, Goldenberg D, Goyal N (2015). Parathyroid localization using 4D-computed tomography. Ear Nose Throat J.

[22] Beland MD, Mayo-Smith WW, Grand DJ, Machan JT, Monchik JM (2011). Dynamic MDCT for localization of occult parathyroid adenomas in 26 patients with primary hyperparathyroidism. AJR Am J Roentgenol.

[23] Chazen JL, Gupta A, Dunning A, Phillips CD (2012). Diagnostic accuracy of 4D-CT for parathyroid adenomas and hyperplasia. AJNR Am J Neuroradiol.

[24] Gafton AR, Glastonbury CM, Eastwood JD, Hoang JK (2012). Parathyroid lesions: characterization with dual-phase arterial and venous enhanced CT of the neck. AJNR Am J Neuroradiol.

[25] Hoang JK, Sung WK, Bahl M, Phillips CD (2014). How to perform parathyroid 4D CT: tips and traps for technique and interpretation. Radiology..

[26] Bahl M, Muzaffar M, Vij G, Sosa JA, Choudhury KR, Hoang JK (2014). Prevalence of the polar vessel sign in parathyroid adenomas on the arterial phase of 4D CT. AJNR Am J Neuroradiol.

[27] Yeh R, Tay YD, Tabacco G, Coronel E, Dercle L (2018) Comparison of sestamibi SPECT/CT and parathyroid 4D CT with a 1-stop simultaneous imaging protocol. J Nucl Med 59:233

[28] Yeh Randy, Tay Yu-Kwang Donovan, Tabacco Gaia, Dercle Laurent, Kuo Jennifer H., Bandeira Leonardo, McManus Catherine, Leung David K., Lee James A., Bilezikian John P. (2019). Diagnostic Performance of 4D CT and Sestamibi SPECT/CT in Localizing Parathyroid Adenomas in Primary Hyperparathyroidism. Radiology.

[29] Fineman I, Johnson JP, Di-Patre PL, Sandhu H (1999). Chronic renal failure causing brown tumors and myelopathy. Case report and review of pathophysiology and treatment. J Neurosurg.

[30] Takeshita T, Tanaka H, Harasawa A, Kaminaga T, Imamura T, Furui S (2004). Brown tumor of the sphenoid sinus in a patient with secondary hyperparathyroidism: CT and MR imaging findings. Radiat Med.

[31] Sutbeyaz Y, Yoruk O, Bilen H, Gursan N (2009). Primary hyperparathyroidism presenting as a palatal and mandibular brown tumor. J Craniofac Surg.

[32] Kar DK, Gupta SK, Agarwal A, Mishra SK (2001). Brown tumor of the palate and mandible in association with primary hyperparathyroidism. J Oral Maxillofac Surg.

[33] Rossi B, Ferraresi V, Appetecchia ML, Novello M, Zoccali C (2014). Giant cell tumor of bone in a patient with diagnosis of primary hyperparathyroidism: a challenge in differential diagnosis with brown tumor. Skeletal Radiol.

[34] Abdel Razek AA (2014). Computed tomography and magnetic resonance imaging of maxillofacial lesions in renal osteodystrophy. J Craniofac Surg..

[35] You M, Tang B, Wang ZJ, Wang KL, Wang H (2018). Radiological manifestations of renal osteodystrophy in the orofacial region: a case report and literature review. Oral Radiol.

